# A modular and self-adjuvanted multivalent vaccine platform based on porcine circovirus virus-like nanoparticles

**DOI:** 10.1186/s12951-022-01710-4

**Published:** 2022-11-24

**Authors:** Ze-Hui Liu, Zhuo-Fan Deng, Ying Lu, Wei-Huan Fang, Fang He

**Affiliations:** 1 Institute of Preventive Veterinary Medicine, Zhejiang Provincial Key Laboratory of Preventive Veterinary Medicine, College of Animal Sciences, Zhejiang University, 866 Yuhangtang road, 310058 Hangzhou, China; 2 Laboratory of Animal Virology of Ministry of Agriculture, Zhejiang University, 310058 Hangzhou, China

**Keywords:** PCV2, VLPs, SpyTag/SpyCatcher, Nanovaccine

## Abstract

**Background:**

Virus-like particles (VLPs) are supramolecular structures composed of multiple protein subunits and resemble natural virus particles in structure and size, making them highly immunogenic materials for the development of next-generation subunit vaccines. The orderly and repetitive display of antigenic epitopes on particle surface allows efficient recognition and cross-link by B cell receptors (BCRs), thereby inducing higher levels of neutralizing antibodies and cellular immune responses than regular subunit vaccines. Here, we present a novel multiple antigen delivery system using SpyCatcher/Spytag strategy and self-assembled VLPs formed by porcine circovirus type 2 (PCV2) Cap, a widely used swine vaccine in solo.

**Results:**

Cap-SC, recombinant Cap with a truncated SpyCatcher polypeptide at its C-terminal, self-assembled into 26-nm VLPs. Based on isopeptide bonds formed between SpyCatcher and SpyTag, classical swine fever virus (CSFV) E2, the antigen of interest, was linked to SpyTag and readily surface-displayed on SpyCatcher decorated Cap-SC via in vitro covalent conjugation. E2-conjugated Cap VLPs (Cap-E2 NPs) could be preferentially captured by antigen presenting cells (APCs) and effectively stimulate APC maturation and cytokine production. In vivo studies confirmed that Cap-E2 NPs elicited an enhanced E2 specific IgG response, which was significantly higher than soluble E2, or the admixture of Cap VLPs and E2. Moreover, E2 displayed on the surface did not mask the immunodominant epitopes of Cap-SC VLPs, and Cap-E2 NPs induced Cap-specific antibody levels and neutralizing antibody levels comparable to native Cap VLPs.

**Conclusion:**

These results demonstrate that this modularly assembled Cap-E2 NPs retains the immune potential of Cap VLP backbone, while the surface-displayed antigen significantly elevated E2-induced immune potency. This immune strategy provides distinctly improved efficacy than conventional vaccine combination. It can be further applied to the development of dual or multiple nanoparticle vaccines to prevent co-infection of PCV2 and other swine pathogens.

## Introduction

Virus infections possess persistent challenges in swine industry leading to severe economic losses worldwide [1]. The economic burden caused by viral infections such as porcine circovirus 2 (PCV2), porcine reproductive and respiratory syndrome virus (PRRSV), swine influenza virus, porcine epidemic diarrhea virus (PEDV), foot and mouth disease virus (FMDV) and many others are associated with severe morbidity, mortality, loss of production, trade restrictions and investments in control and prevention practices [2]. Emerging studies provide evidence that PCV2 has become the predominant problem in major swine producing countries because they are able to affect all stages of production, are highly infectious, have a prolonged shedding duration, and perhaps most importantly, are able to dysregulate the immune response and cause immunosuppression in pigs. Consequently, this situation results in an increased susceptibility to opportunistic infections of viruses and bacteria, leading to multiple infections and substantial economic impact [3–5].

Vaccines can increase the resistance of an animal to infection making them an increasingly important tool in the health management of swine herds. Although many vaccines are efficacious, continual pathogen mutation and the emergence of new virus mutant threats drive a constant need for new and improved vaccines. Safe and highly efficacious live-attenuated vaccines are available against CSFV, but it is difficult to distinguish naturally infected- from vaccinated- animals (DIVA) by means of serological tests of pathogen-specific antibodies, which limit their use during outbreak control or disease eradication programs [6]. To address these challenges, a range of subunit vaccines are being developed based on isolated proteins as protective antigen, which allow DIVA. However, they are often poor immunogens, which require adjuvants to boost their efficacy. As for CSFV E2-based subunit vaccines, they generally induce poor cell-mediated immune responses for sufficient protection efficacy and need two doses [7]. As a consequence, there is a great need to develop novel adjuvants and delivery systems for the next generation of vaccines.

Recently attention has been directed toward the utility of nanoparticles (NPs) as delivery vehicles for vaccines. Amongst some of the first studied NP delivery systems are VLPs; attracting interest because they could facilitate antigen uptake by antigen presenting cells (APCs), increase drug bioavailability and enhance immune responses [8, 9]. VLP-based delivery systems also have the potential to regulate the antigen presentation pathway towards strong cellular immune responses [10–13]. Of note, recombinant PCV2 Cap proteins have been expressed in eukaryotic and prokaryotic expression systems and they assemble spontaneously to form highly organized virus-like particles (VLPs) from 60 Cap subunits. VLPs formed by PCV2 Cap are highly efficacious against PCV2 infection and can be harnessed against PCV2 or/and any pathogen by decorating VLPs with immunostimulatory peptides or epitopes from heterologous pathogens [28]. Using genetic fusion, antigenic epitopes from CSFV, FMDV, influenza A virus or PRRSV was integrated into Cap to generate chimeric PCV2 VLPs as bivalent vaccines [14–18]. However, chimeric PCV2 VLPs only contained small polypeptides from other viruses, and their protective efficacy in vivo remain elusive.

Most importantly, using genetic fusion to integrate foreign epitopes or adjuvants into the VLPs is limited in terms of target proteins of large size and complex three-dimensional structures, which often pose negative effect on VLP assembly, solubility, reasonable protein folding as well as stability [19, 20]. For example, genetically integrating a self-associating protein into VLPs is likely to cause improper protein folding and thereby promote aggregation [21]. A number of strategies have been applied to allow insertion of larger and structurally more complex proteins, using tandem core [22], the split core [23, 24] and mosaic particles technology [25, 26], however steric hindrance is still a limiting factor. An alternative that can bypass this is chemical conjugation via SpyCatcher/SpyTag strategy, which can avoid steric hindrance and achieve targeted antigen display [27].

SpyCatcher is a protein with a size of approximately 15 kDa and contains reactive lysine groups and catalytic glutamate. SpyTag is a long peptide of 13 amino acids, in which the first 10 amino acids contain aspartic acid as a reactive group [28]. In the presence of each other, the carboxyl group of reactive aspartic acid on SpyTag and the ε-amino group of SpyCatcher lysine form a covalent isopeptide bond [29]. One of the main advantages of SpyTag/SpyCatcher technology is that antigens on the surface of VLP can be directed to fully expose functional epitopes, resulting in better antigen processing and subsequent immune response quality [21]. SpyTag/SpyCatcher technology has been reported to display different antigens on self-assembled protein nanoparticles [30, 31] or VLPs formed by HBV surface antigen [32], bacteriophage AP205 [33] or lentivirus [34], and enhances immune activation as demonstrated by *in vitro and in vivo* studies.

Thus, to develop an effective nanoplatform that can simultaneously protect the pigs from the mixed infection of PCV2 and other pathogens, we investigate the potential of PCV2 VLPs to present foreign antigens. For this aim, SpyCatcher was integrated into C terminal of PCV2 Cap to generate chimeric PCV2 VLPs, superfolder green fluorescent protein (sfGFP) or CSFV E2 was genetically fused to SpyTag or SpyCatcher. Based on isopeptide bond formed between SpyTag and SpyCatcher, GFP and CSFV E2 can be readily decorated on the surface of PCV2 VLPs. The coupling efficiency of chimeric PCV2 VLPs and SpyTag or SpyCatcher tagged proteins was then characterized. We further characterize storage stability, the antigen delivery efficiency and adjuvant effect on APCs of this PCV2 VLP-based platform. The efficacy was further evaluated in vivo to evaluate its potential for vaccine development. These data provide novel insights into the immunological properties of nanocarriers used for optimized swine vaccine design.

## Materials and methods

### Design and cloning of expression vectors

Cap-SpyCatcher (Cap-SC) construct includes an N-terminal 6×His tag, PCV2 Cap (amino acids 16 to 233), a flexible linker (GGGGS), and a truncated SpyCatcher [34] at C-terminal. After optimization of *E.coli* codon bias for enhanced protein expression [35, 36], it was synthesized by Hangzhou Tsingke Biotechnology Company. DNA sequence encoding SpyTag (AHIVMVDAYKPTK) was fused to N-terminus of sfGFP (GenBank, ASL68970.1) by PCR to form ST-sfGFP construct (named ST-GFP). E2-Spytag (named E2-ST) construct includes a secretion signal peptide SP23 at N-terminal, CSFV E2ZJ [37], SpyTag and 6xHis tag at C-terminal, and was synthesized by fusion PCR.

Using a one-step cloning kit (Vazyme, cat. C112-01), DNA sequence encoding Cap-SC or ST-sfGFP protein was cloned into *E.coli* expression vector pET28a by in vitro homologous recombination to construct pET28a-Cap-SC and pET28a-ST-GFP. DNA sequence encoding E2-ST was cloned into baculovirus expression vector pFastBac HTA to generate HTA-E2-ST. Recombinant plasmids were identified by PCR and verified by DNA sequencing. The recombinant plasmids pET28a-Cap-SC and pET28a-ST-GFP were transformed into *E.coli* competent cell Transetta (DE3) for subsequent protein expression. According to Bac-to-Bac baculovirus expression system instructions (Invitrogen, cat. 10,359,016), HTA-E2-ST was transformed into DH10Bac, followed by Tn7 transposition, blue-white screening, bacmid extraction and transfection to obtain recombinant baculovirus.

### Expression and purification of Cap-SC or ST-GFP

For large scale expression, 10 mL overnight culture was inoculated into 400 mL LB media containing 100 µg/mL Kanamycin and incubated at 37 °C at 220 rpm. When OD_600_ reached 0.6–0.8, protein expression was induced with the final concentration of 0.5 mM of isopropyl β-d-1-thiogalactopyranoside (IPTG). After incubation at 16 °C for an additional 20 h, bacterial pellet was collected by centrifugation and resuspended in Equilibration buffer (200 mM NaCl, 50 mM Tris-HCl, 10 mM imidazole, pH 8.0) with protease inhibitor cocktail (Transgen, Beijing, China). Bacterial disruption was performed in a high-pressure homogenizer for optimal lysis on ice. Supernatant was collected after centrifugation and filtered through a 0.22 μm filter. After the selective binding of the protein onto a Ni-NTA column (Yeasen, Shanghai, China), elution was achieved by applying a linear gradient of Elution buffer (200 mM NaCl, 50 mM Tris-HCl, 10 mM-500 mM imidazole, pH 8.0). The eluted samples were then evaluated by SDS-PAGE and quantified using the Bradford Assay Kit (Sangon Biotech, Shanghai, China).

### Expression and purification of E2-ST

The expression and purification of E2-ST protein was carried out as described in our previous studies [38, 39]. Purified proteins were dialyzed against Tris-HCl buffer (200 mM NaCl, 50 mM Tris, 10 mM imidazole, pH 8.0), and quantified for following studies.

### Preparation and purification of Cap-E2 NPs and Cap-GFP NPs

Purified ST-GFP or E2-ST were coupled to Cap-SC VLPs in vitro to construct Cap-E2 and Cap-GFP NPs. To explore optimal coupling efficiency, Cap-SC VLPs were mixed with ST-GFP or E2-ST at molar ratios of 1:3, 1:4, or 1:5 overnight at 25 °C. Conjugation efficiency was assessed using SDS-PAGE. For vaccine preparation and purification, Cap-SC VLPs were mixed with ST-GFP at a molar ratio of 1:5 at 25 °C overnight to ensure minimal residual of Cap-SC VLPs at the end of the reaction. Cap-SC VLPs were mixed with E2-ST at a molar ratio of 1:4 at 25 °C overnight to ensure minimal residue of Cap-SC VLPs at the end of the reaction. To assess the percentage of Cap-SC VLPs reacting with ST-GFP or E2-ST, these reactions were analyzed by SDS-PAGE and Coomassie brilliant blue staining, and unreacted Cap-SC VLPs of the same starting concentration were also loaded as a control. ImageJ software was used to analyze the optical density value of each band on SDS-PAGE results. Coupling efficiency was defined as 100×[1–(band of Cap-SC VLPs after coupling reaction)/(band of Cap-SC VLPs before coupling reaction)]. Coupled Cap-GFP NPs and Cap-E2 NPs were purified by superpose 6 size exclusion column to remove unreacted ST-GFP or E2-ST.

### NP characterization

Self-assembly of NPs were analyzed by negative stain transmission electron microscopy (TEM). In brief, samples were placed onto carbon-coated copper grid and adsorbed for 60 s. After removal of excess samples with PBS, grids were stained with 2% (w/v) PTA for 60 s. Negative stained samples were dried and subsequently imaged using a transmission electron microscopy (TEM) fitted with a Gatan OneView camera. Particle diameter and size distribution of NPs were also evaluated by dynamic light scattering (DLS) on a Nicomp™ 380 Particle Sizing system (Santa Barbara, CA, USA) at 25 °C.

### In vitro antigen uptake and immune stimulatory effect on APCs

Mouse bone marrow-derived dendritic cells (BM-DCs) have been used as APC model in studies investigating antigen uptake and presentation in vitro [40–42]. To explore the efficiency of Cap-SC display strategy on antigen uptake, we first compared the difference in internalization efficiency of the soluble protein ST-GFP and Cap-GFP NPs. BM-DCs were prepared according to previous protocol [38, 39], and pre-seeded in six-well culture plates (1 × 10^5^ cells/well) overnight, and then treated with ST-GFP soluble protein or Cap-SC-GFP NPs (equivalent ST-GFP concentration) for 16 h. Green fluorescent signal corresponding to ST-GFP was observed under a confocal laser scanning microscopy. Finally, cells were washed and trypsinized to form a single cell suspension. Green fluorescent signal positive cells then analyzed on a BD FACSCalibur flow cytometer (BD Biosciences, NJ, USA).

Accordingly, the internalization of Cap-E2 NPs was also evaluated in BM-DCs. Pre-seeded cells were incubated with E2-ST soluble protein or Cap-SC-E2 NPs (equivalent E2-ST concentration) for 6 h. After cells were fixed with 4% paraformaldehyde, internalized E2-ST was labeled with CSFV E2 monoclonal antibody 3C12 and FITC-labeled donkey anti-mouse IgG. Finally, the nuclei were stained with DAPI solution (1 µg/mL) for 5 min, and the cellular internalization of E2-ST was recorded by confocal microscopy.

To explore the effect of Cap-E2 NPs on BM-DC maturation and activation, BM-DCs were stimulated with E2-ST or Cap-E2 NPs (same moles of E2-ST), while Cap-SC VLPs (same moles Cap-SC moles) and PBS were set as control groups. After 24 h of incubation, cells were washed and trypsinized to form a single-cell suspension. Subsequently, cells were stained with fluorescent antibodies against CD40 or CD86. The expression of CD40 and CD86 on BM-DCs was determined by flow cytometer. At the same time, cells of each group were collected after the incubation, and subject to total RNA isolation. After reverse transcription, quantitative polymerase chain reaction (qPCR) was performed to analyze the transcription levels of TNF-α, IL-6 and IL-12 cytokines. qPCR primers are shown in Table 1. β-actin was used as an internal control. The relative expression of the target gene was detected by the 2^−ΔΔct^ method.

**Table 1:** The primer sequences used for qPCR

Primer	Sequence (5′–3′)	GenBank ID
β-actin	F: GGAGGGGGTTGAGGTGTT	NM_007393.5
	R: GTGTGCACTTTTATTGGTCTCAA	
TNF-α	F: GCCTCTTCTCATTCCTGCTT	NM_013693.3
	R: TGGGAACTTCTCATCCCTTTG	
IL-6	F: GTTCTCTGGGAAATCGTGGA	DQ788722.1
	R: TCCAGTTTGGTAGCATCCATC	
IL-12 p40	F: GCCAGTACACCTGCCACAAA	NM_008352
	R: TGTGGAGCAGCAGATGTGAGT	

### Animal vaccination experiments

The operating procedures for animal experiments were approved by the Laboratory Animal Management Committee of Zhejiang University (approval number 17,243). Referring to the mouse immune experiment of relevant nanoparticle vaccines [43–46], every effort was made to minimize animal pain, suffering and distress and to reduce the number of animals used. Female BALB/c mice (6–8 weeks old) were randomly assigned to cages with 5 animals per group, and acclimated for 14 days before subsequent experiments. Experimental grouping and antigen dose settings were shown in Table 2 and the vaccine was emulsified with an equal volume of Montanide™ ISA 206 adjuvant (water-in-oil emulsions, SEPPIC, Castres, France). A single dose of each vaccine was used to immunize mice subcutaneously.

**Table 2:** Experimental grouping and antigen dose settings

Group	Test antigen	Total dose (µg)	Cap dose (µg)	E2 dose (µg)
I	Cap-E2 NPs (Cap-SC VLP: E2-SpyTag)	14.6	3.5	10
II	Cap plus E2 (Cap VLP plus E2-SpyTag)	13.5	3.5	10
III	E2 alone (E2-SpyTag)	10	–	10
VI	PBS	–	–	–

### Specific antibodies measurement

According to detection tests of CSFV and PCV2 antibodies established in our previous studies [37, 39, 47, 48], indirect enzyme-linked immunosorbent assay (ELISA) was used to determine serum PCV2- and CSFV-specific antibody. Briefly, recombinant Cap or E2 protein was diluted to 2 µg/mL using carbonate coating solution (pH 9.5), added to ELISA plates at 100 µL/well and incubated overnight at 4 °C. After 3 washes with PBST, 1% caseinate solution was added to block for 2 h at 37 °C. 100 µL of diluted serum samples per well (1:500 dilution in PBST) were added and incubated at 37 °C for 1.5 h. After 3 washes with PBST, 100 µL of diluted HRP-labeled goat anti-mouse IgG (1:5000), IgG1 (1:5000) and IgG2a (1:5000) were added to each well. After incubation at 37 °C for 1.5 h, color reaction was developed with TMB substrate, and analyzed under a microplate reader at 450 nm (OD450). Positive serum and negative serum were also included to verify the reliability and specificity of the test ELISA results.

### Antibody avidity ELISA

Antibody avidity was assessed by its resistance to 8 M urea in binding to specific antigen following previous studies with minor modifications [33, 49]. Briefly, ELISA plates were coated and blocked as described above in duplicate. According to the determination results of IgG in the previous step, the serum samples were adjusted and diluted to OD450 = 1.0 ~ 1.2, and added to two ELISA plates. After incubated at 37 °C for 1.5 h, one of the plates was incubated with 8 M urea (in PBST) for 15 min and then washed 3 times with PBST. Another plate was directly washed 3 times with PBST without 8 M urea. Finally, 100 µL of diluted HRP-labeled goat anti-mouse IgG (1:5000) was added to each well, and the OD450 was read and applied to the relative affinity index, which was defined as (OD450 after 8 M urea treatment)/(OD450 without 8 M urea treatment).

### Virus neutralization test (VNT)

PCV2 JH strain and CSFV Shimen strain-specific neutralizing antibodies were determined in a standard VNT. Serum samples were heat-inactivated at 56 °C for 30 min, followed by 2-fold serial dilutions. 50 µL of viral stock solution (100 TCID_50_) was mixed with an equal volume of diluted serum, added to a 96-well plate and incubated at 37 °C. After incubation for 1 h, virus-serum mixtures were added to PK-15 cell monolayers in triplicate. A serum-free virus mixture was also set up as a control. After incubation at 37 °C with 5% CO_2_ for 3 days, cells were fixed in 4% paraformaldehyde, and virus infection was detected by IFA assay. VNT titers were calculated as the reciprocal of the highest dilution that blocks 50% of viral infection.

### Lymphocyte proliferation assay

Mice were sacrificed 28 days after immunization, the spleens were aseptically collected and homogenized to form a single cell suspension. Spleen lymphocytes were resuspended in RPMI complete medium supplemented with 10% FBS and Penicillin-Streptomycin. Cells were adjusted to a density of 3 × 10^6^ cells/mL, and seeded into 96-well plates (100 µL/well). Recombinant E2 was diluted to 10 µg/mL in RPMI and added to lymphocytes. Concanavalin A (10 µg/mL) and RPMI were set as positive and negative controls. After an additional incubation at 37 °C and 5% CO_2_ for 2 days, 20 µL of MTS solution (5 mg/mL) was added to each well. After 4 h of incubation at 37 °C, OD value was read under a microplate reader. The stimulation index (SI) was calculated according to the formula: SI = (OD value of immune group-OD value of blank control) / (OD value of negative control group-OD value of blank control).

### In vitro cytokine release assay

The lymphocytes prepared in the previous step were seeded into 24-well plates at 1 × 10^6^ cells/well. Cytokine secretion was stimulated at 10 µg/mL E2 protein, RPMI (negative control) and 10 µg/mL concanavalin (Con A, positive control). After further incubation at 37 °C and 5% CO_2_ for 3 days, supernatant was collected and centrifuged to remove cell debris before use. The concentration of IFN-γ, IL-2 and IL-4 in supernatants was measured using Mouse Th1/Th2 Uncoated ELISA Kit (cat. 88-7711-44, Invitrogen). The concentration of Granzyme B (GrB) in these samples was quantified by ELISA Kit (cat. BMS6029TEN, Invitrogen) according to the manufacturer’s instruction.

### Statistical analysis

The experiments were performed with three independent replicates. Data were expressed as the mean ± standard error, and processed with GraphPad Prism 7 (GraphPad Prism Software Inc., San Diego, CA) using t-test. Statistical differences were considered significant if the P-value was less than 0.05 (∗p < 0.05; ∗∗p < 0.01; ∗∗∗p < 0.001).

## Results

### Design and characterization of virus-like particles

**Fig. 1:**
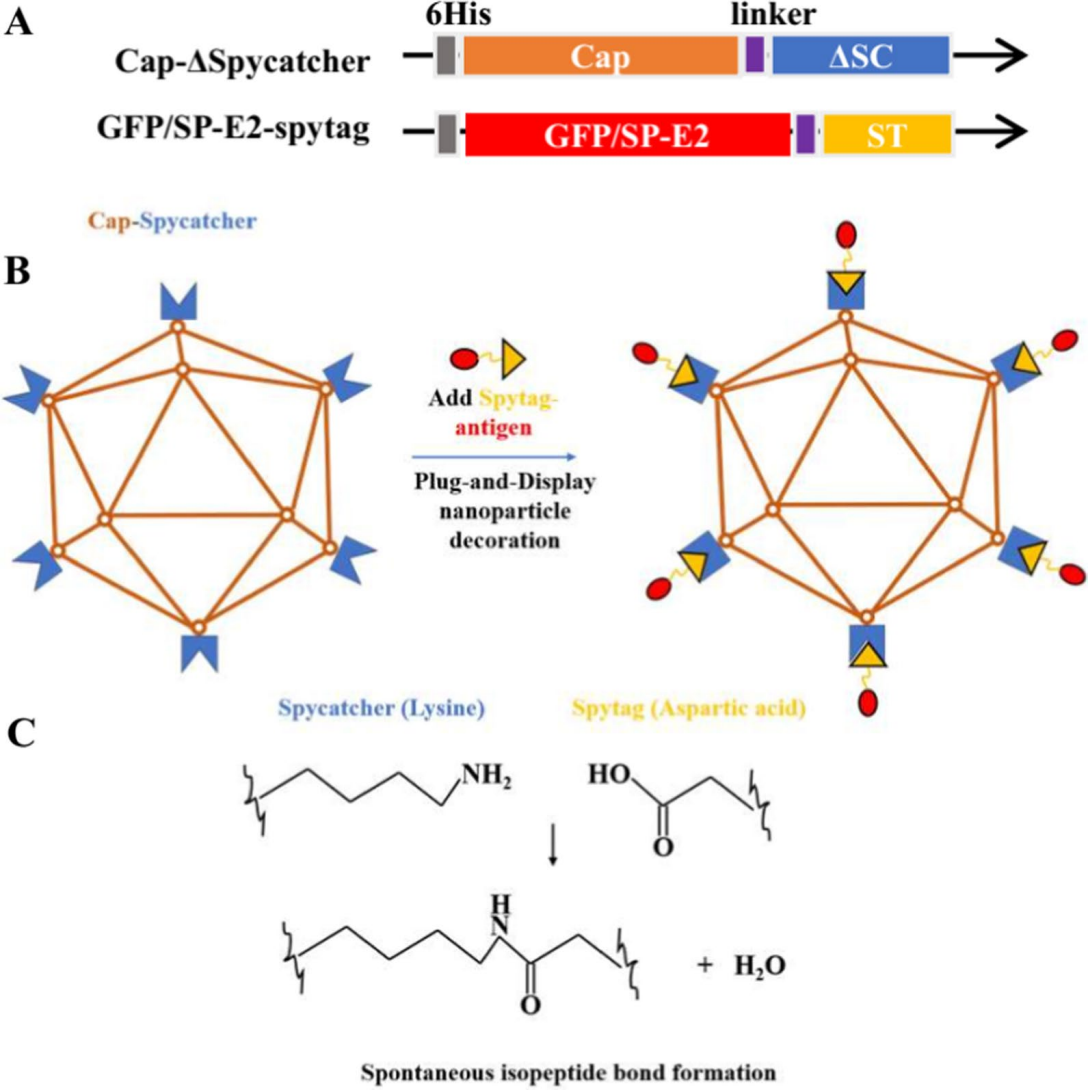
Schematic diagram of Cap-SC VLP based antigen display. **A** vector constructs for expression of Cap-SC protein or ST-tagged GFP or E2. **B** ST-tagged GFP or E2 was coupled and displayed onto Cap-SC VLPs to form Cap-GFP NPs or Cap-E2 NPs. **C** Spontaneous isopeptide formation between the side chains of Asp in SpyTag and Lys in SpyCatcher

**Fig. 2:**
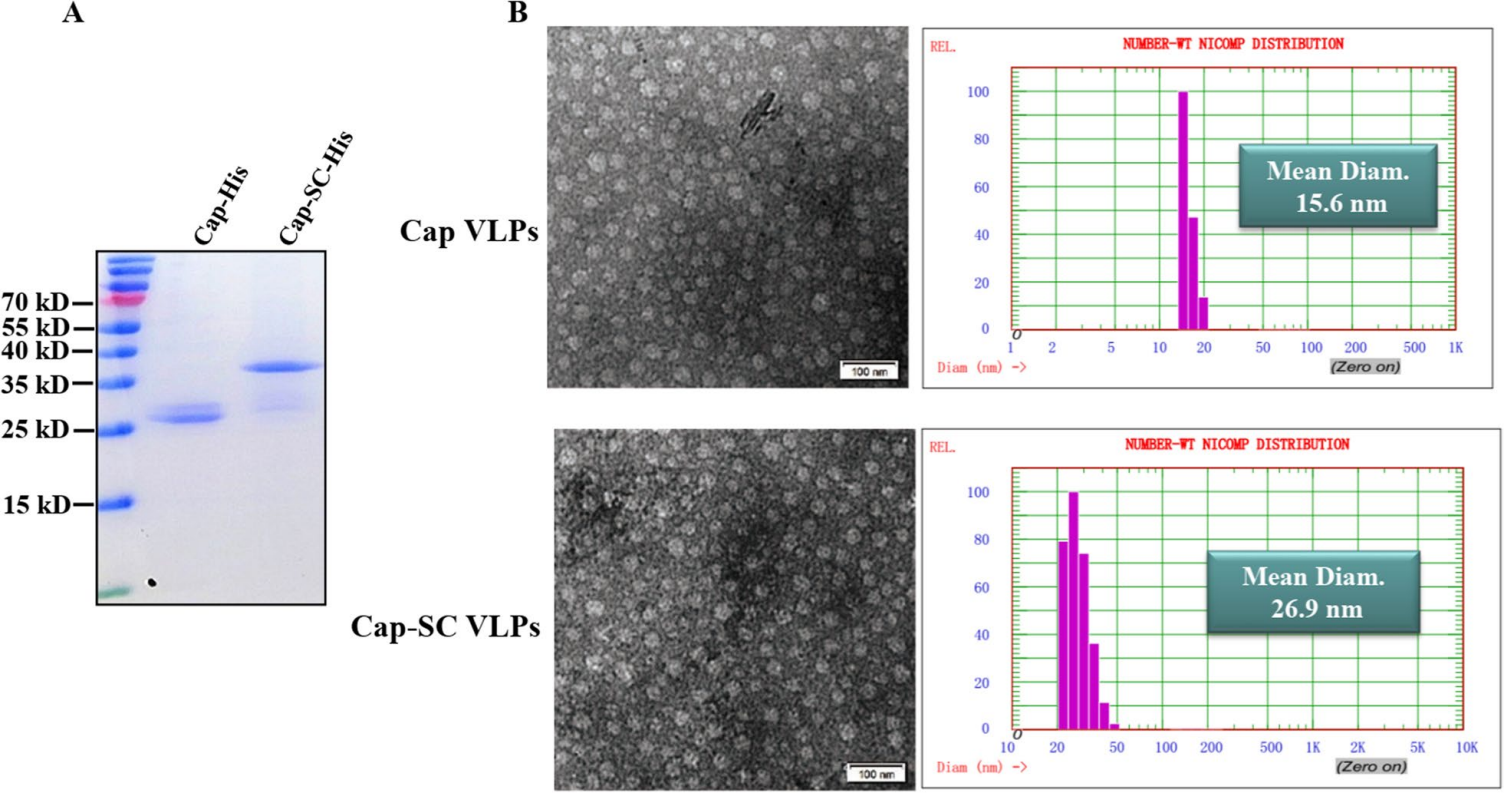
Expression and self-assembly analysis of native Cap or Cap-SC fusion protein. **A** SDS-PAGE analysis of purified Cap fusion proteins. **B** TEM analysis was used to evaluate the self-assembly capacity of Cap fusion proteins. Hydrodynamic diameter and size distribution of Cap VLPs and Cap-SC VLPs were assessed by DLS

PCV2 cap VLP was utilized as antigen delivery platform for nanovaccine development using a Plug-and-Display technology (Fig. 1). In order to evaluate the stability of Cap-SC VLPs for further antigen decoration, a truncated form of SpyCatcher was genetically introduced into C-terminal of Cap, and fusion protein was expressed in *E. coli.* Following protein induction and purification, His-tagged Cap and Cap-SC fusion proteins were of a high purity as shown in SDS–PAGE. Recombinant proteins obtained were of the expected molecular weight (Cap: 27 kD, Cap-SC: 36 kD) (Fig. 2A). These Cap-SC VLPs were stable under room temperature storage for 2 weeks without visual aggregates. In transmission electron microscopy, the produced Cap-SC VLPs showed a similar morphology, but an increased size as compared to native CW2ap VLPs (Fig. 2B). According to dynamic light scattering (DLS), Cap-SC VLPs and Cap-SC were uniform with a narrow size distribution. DLS revealed a homogenous population of non-aggregated particles with an average diameter of 17.5 nm for Cap VLPs and 26.2 nm for Cap-SC VLPs (Fig. 2C). These results revealed that insertion of truncated Spycatcher did not pose any destabilizing effect on Cap self-assembling.

### Decoration of Cap-SC VLPs with ST-tagged antigens

**Fig. 3:**
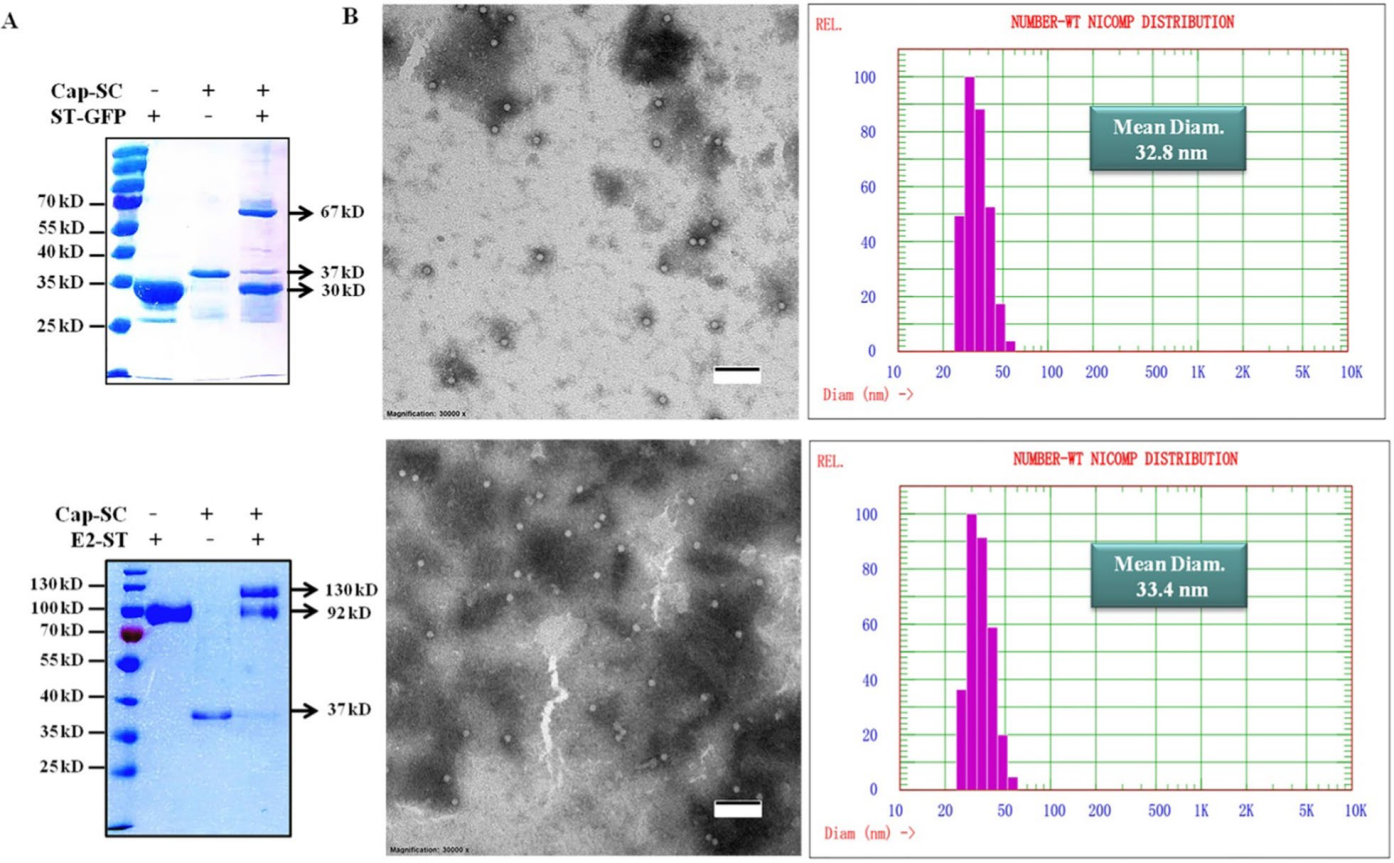
Characterization of Cap-SC VLP-based nanoparticles. **A** Non-reducing SDS-PAGE and coomassie blue staining analysis of Cap-GFP NPs and Cap-E2 NPs. **B** TEM negative staining and DLS analysis of Cap-GFP NPs and Cap-E2 NPs. Scale bar: 200 nm

To explore the antigen-displaying potential of such Cap-SC VLPs, we expressed and purified 2 SpyTag tagged proteins, ST-GFP or E2-ST. After optimizing the molar ratio, Cap-SC VLPs to ST-GFP at a 1:5 molar ratio, and Cap-SC VLPs to E2-ST at a 1:4 molar ratio, were used to increase the reaction efficiency, thus ensuring minimal residual of Cap-SC VLPs. Non-reducing SDS-PAGE showed that after incubation with E2-ST or ST-GFP, the molecular weight of Cap-SC increased significantly after the antigen was conjugated (Cap-GFP NPs:~67 kD, Cap-E2 NPs:~130 kD) (Fig. 3A–B). Further densitometric analysis showed that SpyTag/SpyCatcher reaction can reach 90% coupling efficiency between Cap-SC VLPs and E2-ST, and 84% coupling efficiency between Cap-SC VLPs and ST-GFP. Based on the fact that 60 Cap-SC monomers self-assemble to form 1 VLP, 1 Cap-SC VLP can theoretically bind 60 copies of ST-tagged antigen molecules. Thus, approximately 54 E2 molecules are displayed on each Cap-E2 NP, while approximately 50 GFP molecules are displayed on each Cap-GFP NP. This difference may be attributed to steric hindrance caused by the antigen, resulting in different numbers of SpyCatcher exposed on each Cap-SC VLP.

Subsequently, TEM analysis confirmed an increase in particle diameter of Cap-GFP NPs and Cap-E2 NPs. Likewise, DLS revealed that compared with Cap-SC VLPs (average diameter of 26.2 nm), Cap-GFP NPs had an average diameter of 32.8 nm, and Cap-E2 NPs gave an average diameter of 33.4 nm. This further confirmed surface display of E2-ST or ST-GFP on Cap-SC VLPs (Fig. 3C and F).

### Cap-SC NP mediated antigen delivery in BM-DCs

**Fig. 4:**
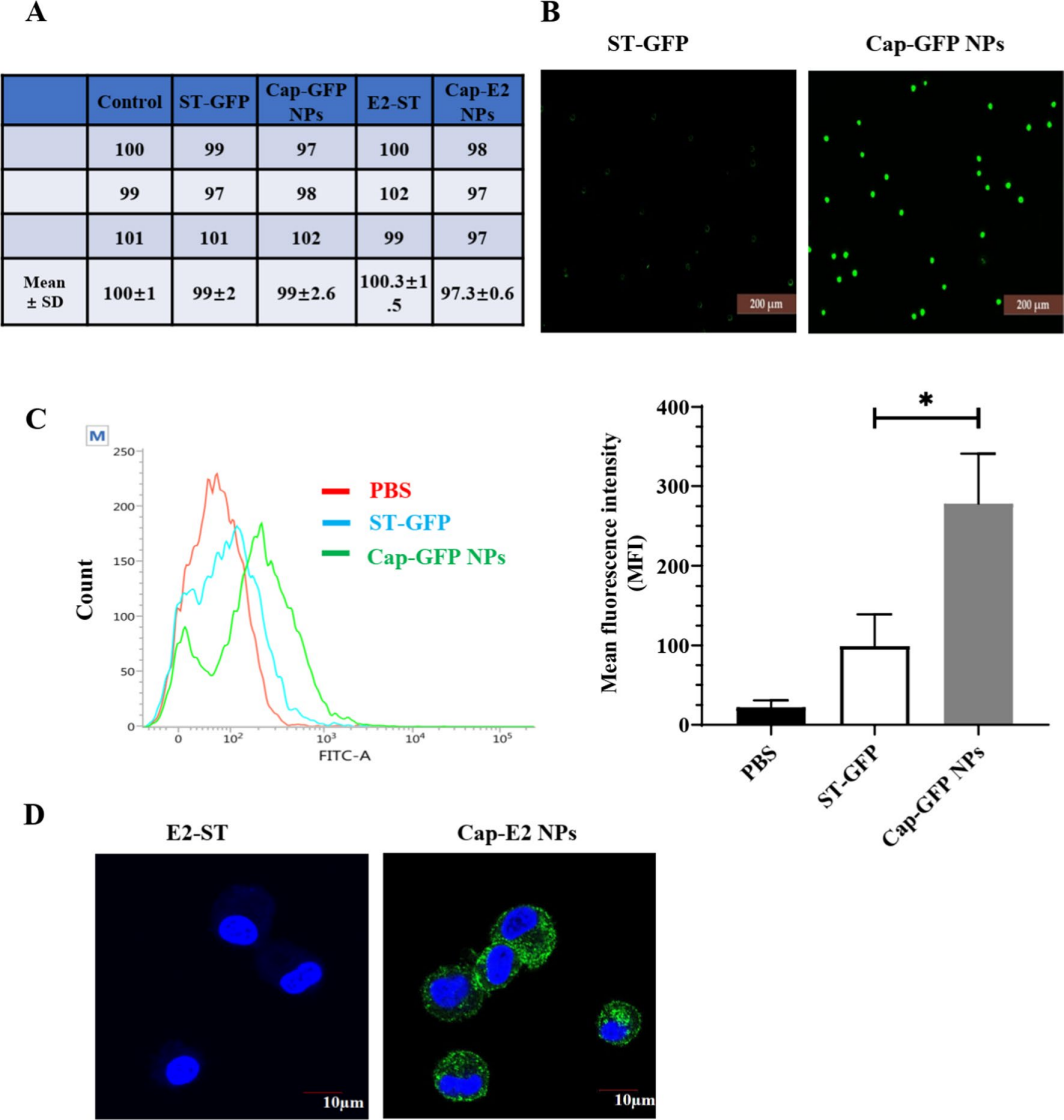
The cellular uptake of Cap-GFP NPs and Cap-E2 NPs by BM-DCs. **A** Cytotoxicity of different proteins on BM-DCs after incubation for 24 h. Data was represented as cell viability (% of the control, Mean ± SD). **B **ST-GFP or Cap-GFP NPs was incubated with BM-DCs for 6 h, levels of internalization were analyzed by inverted fluorescence microscope. **C** levels of internalization were also assessed by flow cytometer and shown as mean fluorescence intensity (MFI). **D** BM-DCs were incubated with E2-ST or Cap-E2 NPs for 6 h, and then subject to immunofluorescence using CSFV E2 monoclonal antibody 3C12 and FITC-labeled donkey anti-mouse IgG. Finally, the nuclei (blue) and E2 (green) signal were recorded by laser confocal microscopy. *p < 0.05

As the most potent APCs, DCs are crucial in inducing protective immunity. The efficiency of antigen uptake by DC greatly affects the magnitude and quality of the immune response. First, CCK-8 experiments confirmed that Cap-GFP and Cap-E2 NPs did not exhibit significant cytotoxicity to BM-DCs cultured in vitro, indicating their excellent biocompatibility (Fig. 4A). To visualize Cap-SC VLP-mediated antigen internalization, Cap-GFP NPs or ST-GFP were incubated with BM-DCs and then analyzed by inverted fluorescence microscopy and flow cytometry. The results showed that Cap-GFP NPs-treated BM-DCs exhibited significantly enhanced GFP fluorescence signal (Fig. 4B). Flow cytometry also indicated stronger antigen internalization in Cap-GFP NPs treated group, of which the mean fluorescence intensity (MFI) was 2.8-fold higher than that of the soluble ST-GFP treated group (P < 0.05) (Fig. 4C).

Similarly, after the incubation with BM-DCs, Cap-E2 NP group showed higher fluorescence intensity compared with the soluble E2-ST group, which was confirmed by IFA and confocal microscopy (Fig. 4D). These results suggest that with the inherent advantages of PCV2 VLPs, antigens displayed onto Cap-SC VLPs can be more efficiently taken up and internalized in BM-DCs.

### Activation and maturation of BM-DCs by Cap-E2 NPs

**Fig. 5:**
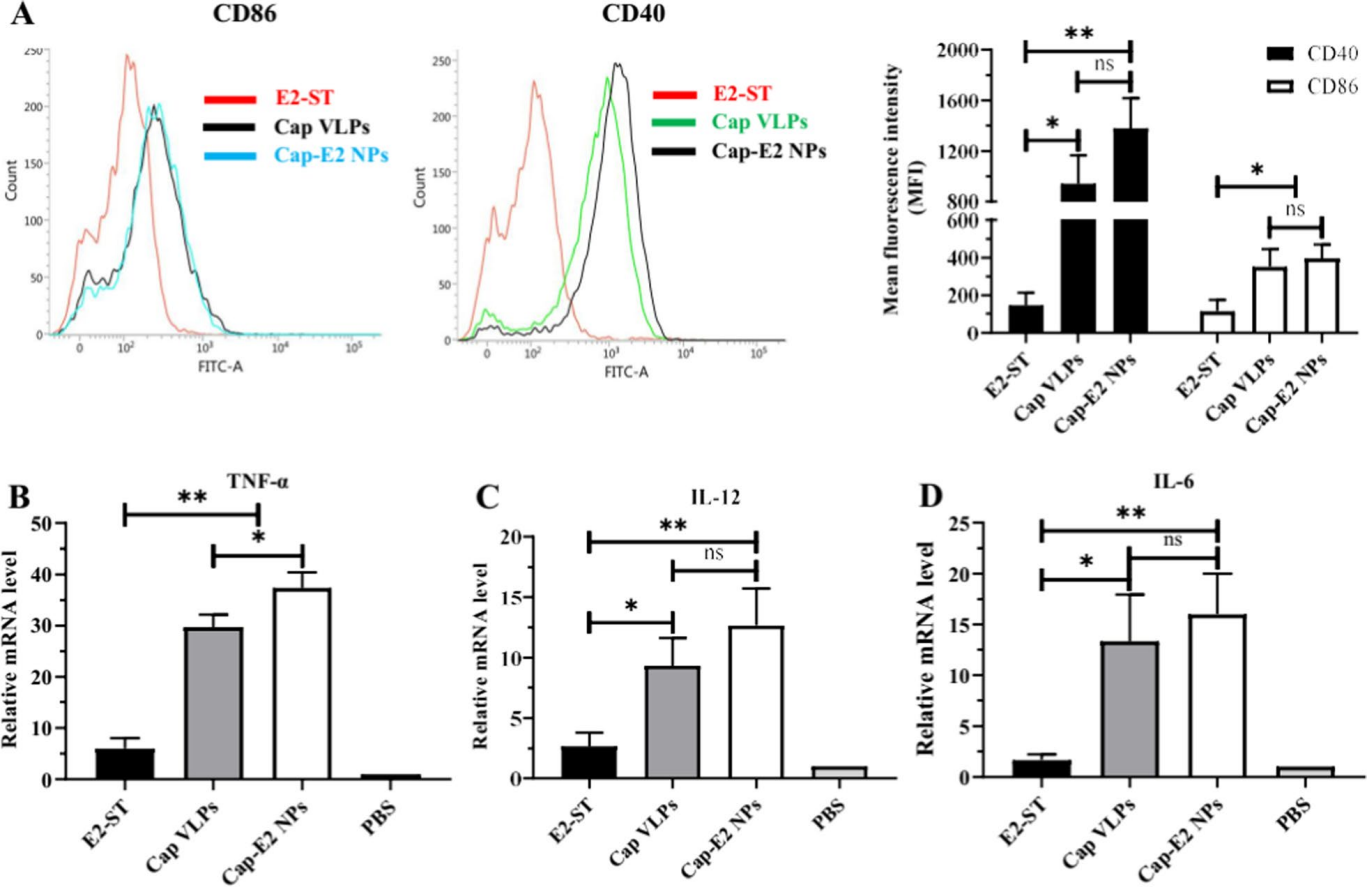
BM-DC costimulatory molecules and cytokines expression induced by Cap-SC based nanoparticles. **A** After incubation with E2-ST, Cap VLPs or Cap-E2 NPs for 6 h, BM-DCs were subject to immunofluorescence using FITC-labelled anti-mouse CD40 or CD86 antibodies, and assessed by flow cytometer and shown as mean fluorescence intensity (MFI). Total RNAs were isolated from E2-ST, Cap VLPs or Cap-E2 NPs treated BM-DCs, and mRNA levels of proinflammatory cytokines TNF-α (B), IL-12 (C) and IL-6 (D) were determined in qPCR, and the data were presented from triplicate experiments. ns, p > 0.05; *p < 0.05; **p < 0.01

After confirming that Cap-E2 NPs can be efficiently captured and internalized in BM-DCs, the effects on BM-DCs activation and maturation were further evaluated. This was mainly achieved by detecting the expression levels of BM-DC surface molecules MHC I/II and costimulatory molecules CD40 and CD86. Flow cytometry results showed that the expression of CD86 and CD40 co-stimulatory markers was significantly elevated in Cap VLPs or Cap-E2 NPs treated groups relative to soluble E2 ST treated groups (Fig. 5A). The mean fluorescence intensity of CD40 in Cap-E2 NPs and Cap VLPs treated groups was 9.2-fold (P < 0.01) and 6.1-fold (P < 0.05) higher than that in E2-ST treated group, respectively. CD86 expression was also up-regulated in Cap-GFP NPs (2.6-fold, P < 0.05) and Cap VLPs (2.4-fold, P < 0.05) treated groups relative to soluble E2-ST-treated groups (Fig. 5A). Since the increased expression of MHC II and CD40 can promote antigen presentation through the MHC II pathway, elevated expression of MHC I and CD86 can enhance antigen cross-presentation via the MHCI pathway. Therefore, it can be concluded that Cap VLPs and Cap-SC based nanoparticles can stimulate DC maturation via enhancing antigen presentation through MHC II pathway and MHC I pathway.

We further investigated whether BM-DCs activation elicits downstream immune responses and promotes cytokine expression. Th1-polarizing cytokines TNF-α and IL-12 act as strong inducers of INF-γ production and effectively enhance protective cellular immune responses. qPCR results showed that relative to soluble E2-ST treated BM-DCs, transcript levels of TNF-α were significantly up-regulated in BM-DCs treated with Cap-E2 NPs (6.2-fold, P < 0.05) and Cap VLPs (4.9-fold, P < 0.05) (Fig. 5B), whereas transcript levels of IL-12 were also significantly enhanced in the BM-DCs treated with Cap-E2 NPs (4.8-fold, P < 0.05) and Cap VLPs (3.7-fold, P < 0.05) (Fig. 5C). Compared with Cap VLPs, slightly up-regulated transcript levels of TNF-α and IL-12 were observed in the Cap-E2 NPs-treated group (TNF-α, P < 0.05; IL-12, P > 0.05). Accordingly, transcript levels of IL-6 were also significantly upregulated in BM-DCs after stimulation with Cap-E2 NPs and Cap VLPs (Fig. 5D), which were 9.2-fold (P < 0.01) and 6.1-fold higher than those treated with soluble E2-ST, respectively (P < 0.05). No significant differences in transcript levels of IL-6 were detected between Cap-E2 NPs and Cap VLPs-treated groups. The inflammatory cytokine IL-6 secreted by DCs is known to induce T cell and B cell activation, proliferation and differentiation. These results indicate that with excellent immune activity of PCV2 VLPs, Cap-SC based nanoparticles can better stimulate the activation of immune cells and promote the generation of subsequent adaptive immune responses.

### PCV2-specific immune responses induced by Cap-E2 NPs

**Fig. 6:**
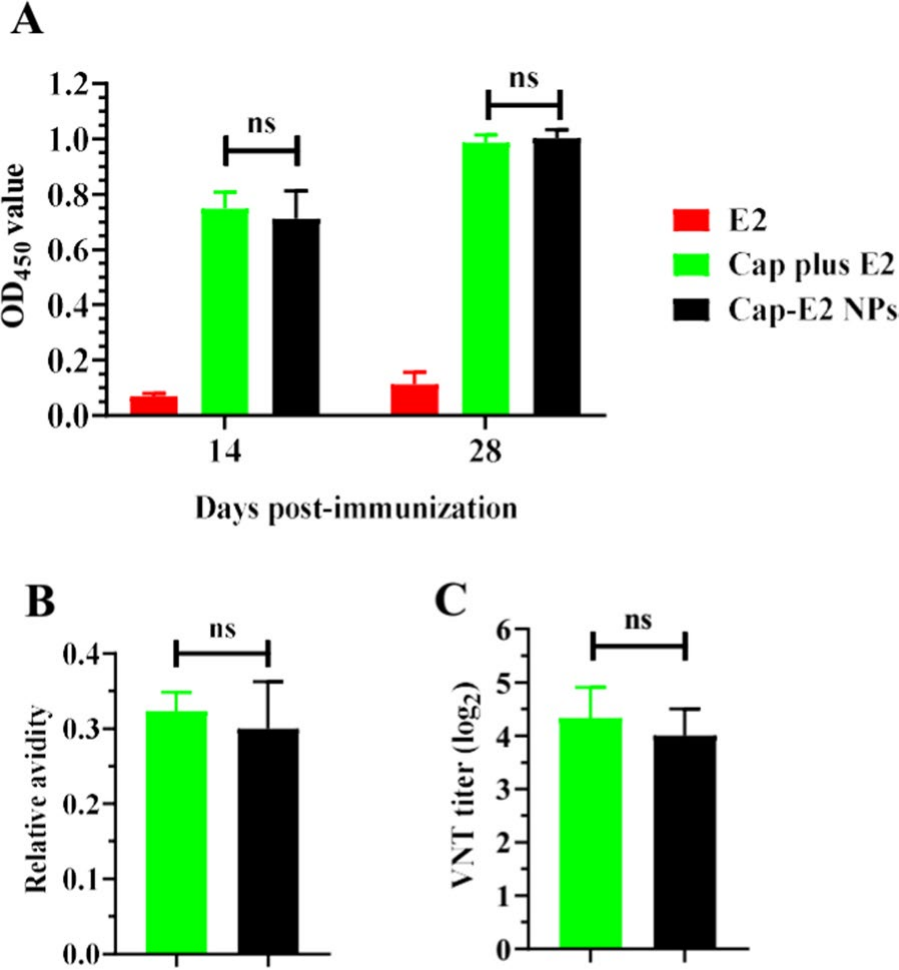
PCV2-specific immune responses induced by Cap-E2 NPs. Mouse sera collected at 14 and 28 dpi (days post-immunization) were further analyzed. **A** indirect ELISA was used to assess binding of serum IgG to native Cap VLPs. **B** The avidity of Cap-specific IgG from mouse sera at 28 dpi was measured by avidity ELISA. **C** Mouse sera at 28 dpi were tested by VNT to quantify PCV2 JH specific neutralizing antibodies. ns, p > 0.05

We first compared Cap-specific IgG levels induced by Cap-E2 NPs and native Cap VLPs using indirect ELISA (Fig. 6A). Control mice inoculated with PBS were negative for Cap-specific IgG. Mice vaccinated with native Cap VLPs or Cap-E2 NPs produced strong Cap-specific IgG responses. Furthermore, using the same amount of Cap protein in either the native Cap VLPs or Cap-E2 NPs immunized groups (Table 2), no significant difference in Cap-specific IgG responses between the Cap VLPs and Cap-E2 NPs groups were detected at both 14 dpi and 28 dpi (P > 0.05). Likewise, avidity ELISA showed that IgG induced by native Cap VLPs had a mean relative avidity, which was higher than that induced by Cap-E2 NPs (Fig. 6B).

Neutralizing antibody levels were detected by measuring the blocking activity of immune sera against the homologous PCV2d JH strain. As shown in Fig. 6C, VNT titers in the native Cap VLPs group were slightly higher than those in the Cap-E2 NP group at 28 dpi, but did not reach significant difference (P > 0.05). Sera from mice immunized with PBS did not display any neutralizing activity. Based on these results, display of SpyCatcher on Cap VLPs and subsequent conjugation of E2 antigen on VLP via SpyCatcher/SpyTag technology had no significant effect on ability of VLPs to induce PCV2-specific humoral immune responses.

### Enhanced CSFV-specific humoral immune responses induced by Cap-E2 NPs

**Fig. 7:**
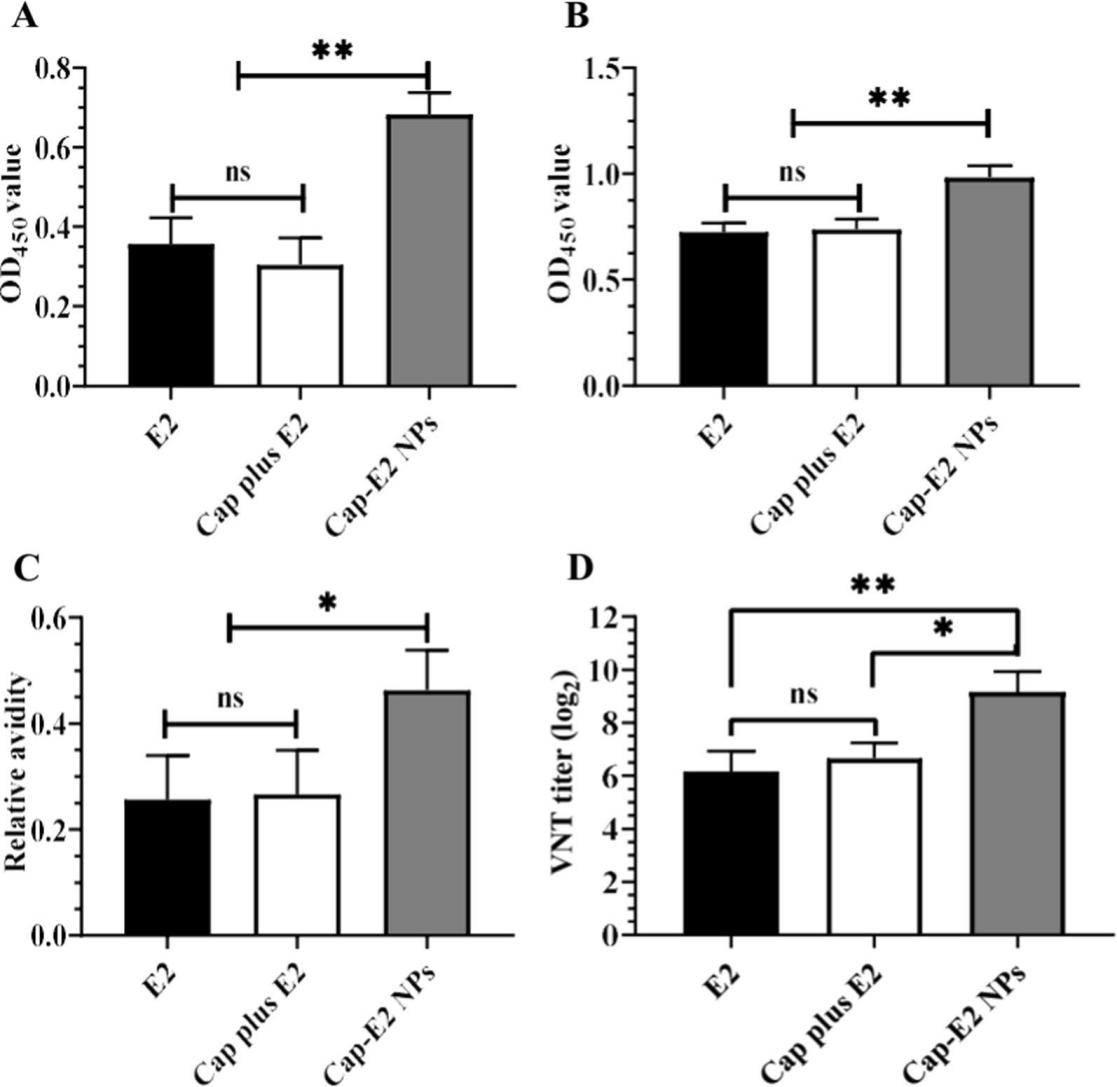
CSFV-specific humoral immune responses induced by Cap-E2 NPs. Serum samples were collected for detection of CSFV E2 specific antibodies at 14 dpi (**A**) and 14 dpi (**B**) with indirect ELISA. **C** The avidity of the E2-specific IgG from mice sera at 28 dpi was measured by avidity ELISA. **D** A standard VNT was performed to evaluate neutralizing antibodies against CSFV Shimen strain. ns, p > 0.05; *p < 0.05; **p < 0.01

To investigate induction of antibodies directed against E2 displayed on Cap VLPs, the sera of mice immunized with Cap-E2 NPs were assayed for the presence of E2-specific total IgG and IgG subtype antibodies. After a single dose vaccination, sera were collected at 14 dpi and 28 dpi for indirect ELISA analysis. The results showed that Cap-E2 NPs immunization induced the highest E2-specific IgG levels both at 14 dpi and 28 dpi, which were significantly higher than Cap plus E2 group (Cap VLP mixed with E2-SpyTag) and E2 alone group (E2-SpyTag) (Fig. 7A–B, p < 0.01). No significant difference was observed between the Cap plus E2 group and the E2 alone group. In addition to E2-specific IgG levels, we also assessed the relative affinity of E2-specific IgG at 28 dpi. As shown in Fig. 7C, the relative affinity of E2-specific IgG was significantly increased in the Cap-E2 NPs immunized group compared to the groups with Cap plus E2 or E2 alone (p < 0.05).

Since neutralizing antibodies play an important role in CSFV immune protection in vivo, we further determined VNT titers against virulent CSFV Shimen strain. Compared with PBS control group, VNT titers were significantly increased in all immunized groups, with an average titer of 1:572 in the Cap-E2 NPs group, 1:101 in the group of Cap plus E2, and 1:72 in the E2 alone group (Fig. 7D). VNT titers in the group of Cap-E2 NPs were significantly higher than those in Cap-plus-E2 group (p < 0.05) and E2-alone group (p < 0.01). These results indicated that when displayed on Cap-SC VLPs, E2 was more immunogenic and potent to evoke CSFV-specific humoral immunity.

**Fig. 8:**
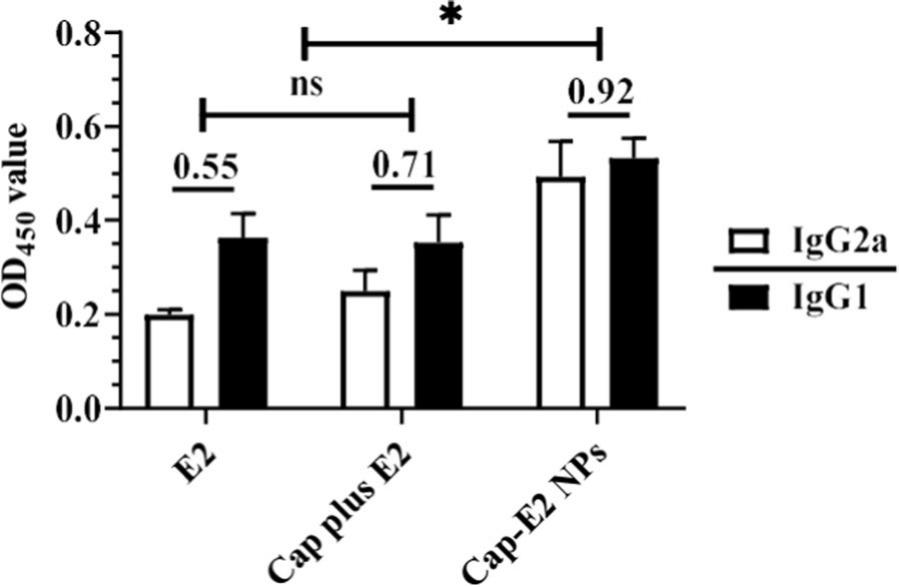
CSFV-specific IgG2a and IgG1 induced by Cap-E2 NPs. Serum samples were collected at 28 dpi for detection of CSFV E2 specific IgG2a and IgG1 with indirect ELISA. ns, p > 0.05; *p < 0.05

IgG subtype switching is known to be regulated by Th cells (IgG2a secretion is mediated by Th1 cells; IgG1 secretion is mediated by Th2 cells). We therefore explored the effect of Cap-E2 NPs immunization on Th1 and Th2 cell polarization. ELISA results showed that the IgG2a/IgG1 ratio of Cap-E2-NP group was significantly higher than that of Cap-plus-E2 group and E2-alone group (Fig. 8, p < 0.05). The higher IgG2a/IgG1 ratio indicated that Cap-E2 NPs potentiated Th1 polarization, which was believed to further enhance the cellular immune response.

### Improved CSFV-specific cellular immune responses induced by Cap-E2 NPs

**Fig. 9:**
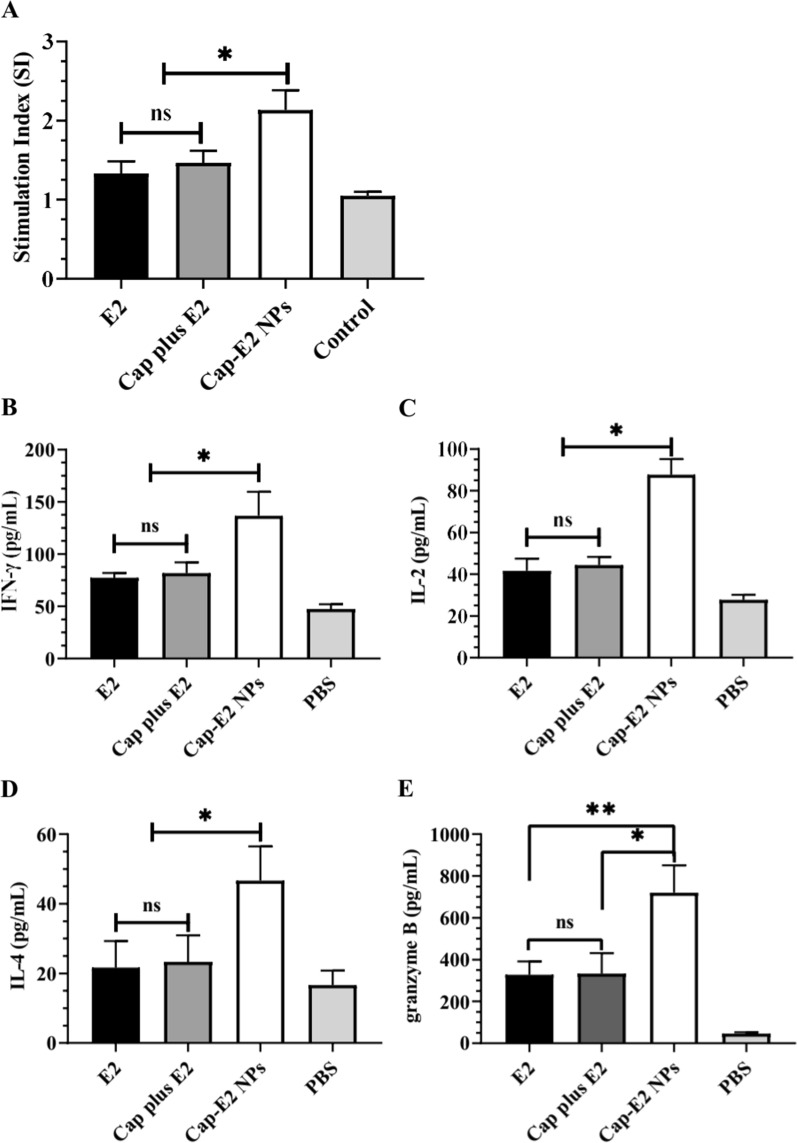
Detection of CSFV cellular immunity related factors. Spleen lymphocytes were isolated from the immunized mice and stimulated with CSFV E2 in triplicate. **A** stimulus index (SI) was analyzed using MTT method. The supernatants were tested for the presence of IFN-γ (**B**), IL-2 (**C**), IL-4 (**D**) and GrB (**E**). ns, p > 0.05; *p < 0.05; **p < 0.01

To evaluate the superiority of Cap-E2 NPs in inducing CSFV-specific cellular immunity, we isolated spleen lymphocytes from each immunized group for analysis of lymphocyte proliferation activity, cytokine release, and antigen-specific cytotoxic T-lymphocyte (CTL)-mediated cytotoxicity on infected cells (CTL activity). As shown in Fig. 9A, the proliferation assay showed that stimulation index (SI) in the group of Cap-E2 NPs was significantly enhanced upon stimulation with recombinant E2 antigen (compared with the group of Cap-plus-E2, p < 0.05; compared with E2 alone, p < 0.01). No significant differences were detected between Cap-plus-E2 group and E2-alone group. SI in the group of Cap-plus-E2 was slightly increased relative to the group of E2 alone, which may be caused by the adjuvant effect of Cap VLPs.

ELISA assay showed that all immunized groups induced significantly higher levels of Th1 cytokines IFN-γ and IL-2 than the PBS control group (Fig. 9B-C). Cap-E2-NP group produced the highest levels of IFN-γ and IL-2 upon stimulation with recombinant E2 antigen (p < 0.05 vs. Cap plus E2; p < 0.05 vs. E2 alone). In terms of Th2 cytokine IL-4, Cap-E2 NPs group showed a significant increase, while IL-4 secretion was not markedly increased in the groups either with Cap-plus-E2 or E2 alone relative to the PBS control group. Cap-E2 NPs induced higher IL-4 levels than Cap plus E2 group and E2 alone group, with a significant difference (Fig. 9D, p < 0.05). These data are consistent with the elevated IgG2a/IgG1 ratio, which suggests a trend towards induction of Th1 polarization in the group of Cap-E2 NPs.

As one of the most important CTL mediators, granzyme B is often used to judge the activity of CTLs that specifically recognize and kill virus-infected cells. As shown in Fig. 9E, the release of granzyme GrB in response to recombinant E2 antigen was significantly increased in all immunized groups compared to the PBS control group. The secretion level of granzyme GrB in Cap-E2 NPs group was 2.1-fold and 2.2-fold higher than Cap plus E2 group (p < 0.05) and E2 alone group (p < 0.01), respectively.

## Discussion

Self-assembled protein-based nanoparticle platforms have been exploited to deliver heterologous antigens, emerging as a trending direction for the development of next-generation subunit vaccines. Nanoparticle generated from hepatitis B surface antigen (HBsAg) VLPs displaying *P. falciparum* T cell epitopes have recently been approved for clinical use as an effective malaria vaccine [50]. Although the potential of various self-assembling protein nanoparticles as vaccine vehicles has received extensive attention, the applicability of VLPs formed by PCV2 Cap as antigen carriers has not been intensively investigated. Surface-display of exogenous antigen inserted into Loop CD domain [51, 52] or C-terminus of Cap [17, 53] has provided supporting evidence of PCV2 VLPs to act as a vaccine platform. However, according to the resolved Cap structure, the integration of full-length exogenous protein into Cap may impair VLP assembly, stability, solubility, and correct antigen folding, so these vaccines are limited to the introduction of small epitopes/peptides to generate chimeric Cap VLPs [54]. They can induce specific immune responses directed against these displayed epitopes/peptides, but fail to elicit sufficient protective immune responses comparable to full-length protective antigens [15, 16, 51]. In addition, although PCV2 VLPs are effective vaccine candidates to prevent PCV2 infection and related diseases, PCV2 is often co-infected with other important swine viruses and bacteria, thus aggravating clinical symptoms and complicating disease prevention and control [55, 56]. Therefore, the development of bivalent nanoparticle vaccines by displaying full-length protective antigens of heterologous pathogens on the surface of PCV2 VLPs can facilitate the vaccination against PCV2 and co-infected pathogens.

In this study, we combined PCV2 VLPs and SpyCatcher/SpyTag technology to establish an antigen delivery system. Given the fact that C-terminus of PCV2 Cap is surface exposed and compatible to the insertion of exogenous polypeptides, SpyCatcher was introduced into C-terminus of PCV2 Cap by genetic fusion. Therefore, PCV2 Cap-SC fusion protein could theoretically self-assemble into VLPs displaying 60 copies of SpyCatcher on its surface. In this way, this nanoscaffold provides 60 binding sites for functional protein display. As expected, TEM and DLS analysis showed that Cap-SC VLPs had good stability, showing a homogeneous structure and narrow size distribution. Furthermore, the full-length ST-GFP and E2-ST proteins were displayed onto Cap-SC VLPs at a high density, with 50 copies of ST-GFP or 54 copies of E2-ST per VLP.

To date, accumulating studies revealed that particle vaccine size strongly affects vaccine properties [57–59]. Rational design of vaccines of specific sizes will enable coordinated immune responses. VLPs and VLP-derived nanoparticles in vaccines closely resemble native viruses. With optimal size (20–200 nm), these nanoparticles have been engineered to deliver less immunogenic subunit vaccine antigens, and enable the preferential uptake of antigens by antigen-presenting cells (APCs), thus lead to more effective activation of APC and subsequent adaptive immune responses [60, 61]. Accordingly, our results indicated that Cap-SC VLPs derived Cap-GFP NPs (32.8 nm) or Cap-E2 NPs (33.4 nm) could be more efficiently recognized and internalized by BM-DCs. This enhanced effect may be due to Cap-GFP NPs or Cap-E2 NPs mimicking native PCV2 Cap VLPs, which are internalized by APCs via clathrin-dependent endocytosis [62].

After antigen internalization, DCs undergo a series of events often referred to as DC maturation, antigen presentation to CD4^+^ or CD8^+^ T cells through MHC class II or MHC class I pathways, and the secretion of immunostimulatory cytokines [38, 39]. Our data show that costimulatory molecules CD40 and CD86 expression were elevated in BM-DCs upon stimulation with Cap-E2 NPs and Cap VLPs. Moreover, Cap-E2 NPs or Cap VLPs, instead of soluble E2, significantly enhanced the release of Th1-polarizing cytokines (TNF-α and IL-12) and Th2-polarizing cytokine IL-6. DC maturation was characterized by upregulated expression levels of costimulatory molecules and surface markers [63]. Both IL-12 and TNF-α are necessary to stimulate T-cell proliferation and induce strong antigen-specific CTL responses, whereas IL-6 can promote T and B cell activation, proliferation, and differentiation [64, 65]. Taken together, these results indicated that inherent advantage of Cap VLP-based vaccine delivery could significantly accelerate the maturation and activation of DC and markedly enhance the synergistic immune responses in vitro.

Surface antigen density is critical for B-cell receptor (BCR) cross-linking and B-cell activation, which account for higher neutralizing antibody response. Based on the SpyTag/SpyCatcher technology, researchers have developed multiple SARS-CoV-2 nanoparticle vaccines. These vaccine strategies include RBD-Ferritin (24-mer) prepared by displaying 24 copies of the receptor binding domain (RBD) on Ferritin nanoparticles, RBD-mi3 (60-mer) prepared by displaying 60 copies of RBD on mi3 nanoparticles and RBD-I53-50 (120-mer) prepared by displaying 120 copies of RBD on I53-50 nanoparticles [30, 31, 66]. Antigens displayed at high density on these nanoparticles induced significantly enhanced neutralizing antibody responses and cellular immunity compared with monomeric antigens, and only a single-dose immunization with these nanoparticle vaccines achieved reinforced protection against SARS-CoV-2 infection in mouse [67, 68]. Here, our results also provided supporting evidence that high-density display of antigens on nanoparticles has great potential to boost antigen specific immune responses. Cap-E2 NPs induced significantly higher antibody levels and neutralizing antibody responses than unconjugated vaccines (Cap plus E2 and E2 alone), while there was no significant difference between unconjugated vaccine (Cap plus E2) and E2 alone group. This indicates that, as expected, E2 was more immunogenic when it was displayed on the VLP surface. In addition, Cap-SC conjugated E2 NPs elicited antibodies with dramatically higher binding avidities. This is consistent with a previous report where Pfs25 displayed on AP205 VLPs elicited significantly higher antibody level, and the antibodies also had stronger avidity than that of monomeric Pfs25 antigen [33].

Numerous studies have shown that nanoparticle-based vaccines can help to enhance cross-presentation efficiency and priming of CD8^+^ T cell responses, which is pivotal for vaccination against cancer and infection by intracellular virus and bacteria [69]. Cellular response to intracellular pathogens could be characterized by differentiation of CD8^+^ T cells into CTLs, which killed infected cells *via* granzyme B secretion; and/or differentiation of CD4^+^ Th0 cells into CD4^+^ Th1 cells, which secrete cytokines (such as IFN-γ) to exert a direct antiviral effect [65]. Encouragingly, our results showed that E2 displayed on Cap-SC VLPs through SpyCatcher/SpyTag conjugation resulted in significant induction of CSFV E2 specific cellular immunity. Elevated lymphoproliferative responses and more Th1 type cytokine (IL-2 and IFN-γ) production were detected in the mice immunized with Cap-E2 NPs instead of unconjugated vaccine (Cap plus E2 and E2 alone). Furthermore, Cap-E2 NPs evoked a potent CTL response to E2, as evidenced by more antigen-specific GrB secretion (an important mediator of the CTL), which is involved in direct killing of infected cells.

IgG subclass expression reflects the subset of CD4^+^ Th cells (Th1 and Th2) polarized in immune responses leading to the different mechanisms of host protection process [70]. Th2 cytokine IL-4 mainly induces switching to IgG1, whereas IFN-γ induces switching to IgG2a [71, 72]. IgG1 is normally the most abundant subclass and dominated with antibody mediated responses. IgG2a is associated with clearing virus infections. Among IgG subclasses, IgG2a and 2b are generally considered to be the most potent for activating immunity responses and dominating antiviral immunity and autoimmune diseases [73–75]. IgG1 corresponds to Th2-biased responses, while IgG2a corresponds to Th1-biased responses. The ratio of IgG2a/IgG1 indicated that Cap-E2 NPs induced cellular immune response directed towards Th1-biased response in some degree. The combination of IgG2a and Th1 type cytokines (IFN-γ) suggest a better cellular immune response elicited by Cap-E2 NPs.

As vaccine-induced neutralizing antibodies and cell-mediated immunity are generally considered a correlate of protective immunity against CSFV [6, 76], our findings highlighted the potential of this Cap-SC VLP based vaccine to increase functional antibody responses as wells as cellular immune responses against displayed antigens. Of note, we found that the display of SpyCatcher on the surface of Cap VLPs by genetic fusion, and the subsequent assembly of CSFV E2-ST antigen onto the surface of Cap-SC VLPs via SpyTag/SpyCatcher did not significantly interfere with the immunogenicity of Cap VLPs. Cap-E2 NPs and native Cap VLPs induced equivalent levels of PCV2-specific and neutralizing antibodies. This may be benefit from the large surface area of the PCV2 VLP icosahedron, while the surface-modified SpyCatcher and E2 protein did not mask the major antigenic and neutralizing epitopes. Numerous previous studies have shown that PCV2 neutralizing antibody levels are positively correlated with protective efficacy against PCV2 challenge, and that high levels of neutralizing antibodies can significantly reduce PCV2 load and effectively prevent PCV2-related clinical diseases [77–80]. This suggests that Cap-E2 NPs can also induce comparable levels of PCV2-specific protective efficacy.

This modular approach is particularly attractive for vaccine production against rapidly changing pathogens, such as influenza, where the displayed antigens may require periodic modification. Neutralizing antibodies targeting HA (especially the head region) effectively prevent viral infectivity by blocking the interaction between sialic acid receptors and host cells. However, HA proteins frequently undergo point mutations that enable them to evade host immune surveillance, and thus vaccines must be reformulated annually against seasonal influenza viruses with new antigenic variants. The modular vaccine strategy we developed allows for timely vaccine updates against swine influenza while preventing and controlling PCV2 infection. It will help in effective control of swine influenza and reducing the probability of IAV cross-species reassortment and transmission. Further studies are ongoing to evaluate the potential of this PCV2 VLP based nanoparticle to vaccinate against swine influenza.

## Conclusion

In conclusion, our data shows that Cap VLPs can be exploited as a platform to deliver foreign antigens in the form of full-length proteins while exhibiting adjuvant-like effect and excellent self-immunogenicity. Cap-E2 NPs made with this strategy resulted in a more robust immune response against CSFV E2 with improved performance in antibody titer, binding avidity, subclass distribution and protective cellular immunity. This immunogenic response was significantly higher than the soluble E2 or the admixture of Cap VLP and E2. These findings highlight the feasibility of making dual vaccines by coupling ST-tagged protective antigen of a variety of swine pathogens to Cap-SC VLPs, which represent a modular vaccine strategy applicable to accelerate vaccination against a range of diseases, including emerging and re-emerging pathogens such as swine influenza virus.
